# The relationship between metabolic syndrome and obstructive sleep apnea syndrome: a nationwide population-based study

**DOI:** 10.1038/s41598-021-88233-4

**Published:** 2021-04-22

**Authors:** Do Hyun Kim, Bongseong Kim, Kyungdo Han, Soo Whan Kim

**Affiliations:** 1 Department of Otolaryngology-Head and Neck Surgery, Seoul St. Mary’s Hospital, College of Medicine, The Catholic University of Korea, Banpo-daero 222, Seocho-gu, Seoul, 137-701 Republic of Korea; 2 Department of Statistics and Actuarial Science, Soongsil University, 369 Sangdo-ro, Dongjak-gu, Seoul, 06978 Republic of Korea

**Keywords:** Diseases, Endocrinology

## Abstract

There has been a need for research on the association between metabolic syndrome (MetS) and obstructive sleep apnea syndrome (OSAS) using large data such as nationwide population-based data that adjusts important confounding factors. Therefore, we investigated the relationship between MetS and OSAS. The data source we used was the National Health Insurance Service claims database managed by the Republic of Korea government, in which 10,113,560 individuals were enrolled in 2009 and followed up until 2018. The independent association of MetS with the risk of OSAS was determined using a Cox proportional hazards model with adjustment for age, sex, smoking status, alcohol consumption, regular physical exercise, and body mass index. Our results showed that MetS were strongly associated to OSAS which was adjusted for several confounding factors. Also, we found men, increased waist circumference and increased triglyceride are important risk factors for OSAS.

## Introduction

Metabolic syndrome (MetS) comprises multiple cooccurring conditions that increase the risk of diabetes and cardiovascular disease, including abdominal obesity, high blood pressure, hyperglycemia, and dyslipidemia^1–3^. MetS was diagnosed based on the criteria proposed by the International Diabetes Federation, i.e., three of more of the five MetS components: high blood pressure (SBP ≥ 130 mmHg, DBP ≥ 85 mmHg) or treatment for previously diagnosed hypertension, elevated triglyceride (TG) level (≥ 150 mg/dL), low high-density lipoprotein cholesterol (HDL-C) level (< 40 mg/dL for men and < 50 mg/dL for women), elevated fasting plasma glucose level (≥ 100 mg/dL) or previously diagnosed type 2 DM, and abdominal obesity (waist circumference ≥ 90 cm for men and ≥ 85 cm for women)^4^. Up to one billion middle aged people have OSAS worldwide^5^, and the number of OSAS patients has tended to increase along with the obesity epidemic^6–8^. Some conditions not classified as MetS components may nevertheless exacerbate cardiovascular disease in MetS patients, such as obstructive sleep apnea syndrome (OSAS). OSAS is considered one of the most significant sleep disorders, with a prevalence of 9–17%^9^. It is a chronic respiratory condition, in which the air flow is repeatedly blocked during sleep, resulting in recurrent hypoxia, hypercapnia, and arousal^10,11^. OSAS can be both a sleep disorder and a heterogeneous metabolic disorder^12^. Meta-analyses have shown an association between MetS and OSAS^13,14^; however, many factors modulate the association of MetS with OSAS, making it difficult to assess the relationship between them with precision^15–17^; Also, no report on the association between the two diseases has adjusted for body mass index (BMI)^17^. Also, it has been suggested that to investigate the association between MetS and OSAS, a study including a large population and adjusting for confounding factors is needed^15^.

Therefore, we conducted this study to evaluate the association between MetS and OSAS using nationwide population-based data. In addition, because the incidence of OSAS differs by gender, we also conducted analyses stratified according to this variable.

## Materials and methods

### Data sources and study population

**Figure 1:**
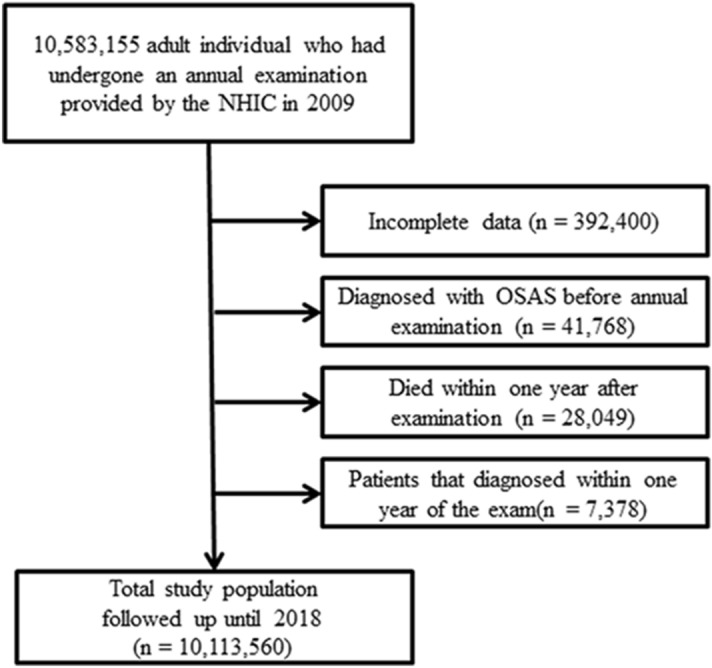
Flow chart of the study population.

Our data source was the National Health Insurance Service (NHIS) claims database (NHIS-2020-1-313), managed by the Republic of Korea government. We analyzed data for the period 2009–2018. In Korea, the NHIS offers health insurance to 97% of the population^18^. This study was approved by the Ethics Committee of the National Evidence-Based Healthcare Collaborating Agency, and the need for informed patient consent was waived. The NHIS reviews claims from both outpatients and inpatients, and holds data on diagnoses, procedures, prescriptions, and demographics. Claims from the Medical Assistance Program and the Medical Care for Patriots and Veterans Affairs Scheme, which cover medical expenses not reimbursed by the NHIS, are also reviewed by the latter body. Therefore, the entire Korean population is well represented by the NHIS database, which rules out selection bias. Because the NHIS deidentifies personal information to ensure anonymity, the institutional review board of Seoul Saint Mary's Hospital waived the requirement for informed consent for this study (KC19ZESI0560). All procedures were carried out in accordance with the Helsinki Declaration of 1964 and subsequent amendments or equivalent ethical standards. In 2009, 10,583,155 adults (aged > 19 years) were examined through annual checkups provided by the NHIC. Due to incomplete data, 392,400 cases were excluded from our analysis. To accurately calculate the incidence of OSAS, 41,768 patients diagnosed with OSAS before the annual examination, and 28,049 who died within 1 year of the examination, were also excluded, as were 7378 patients diagnosed within 1 year of the exam to preclude any effect of time differences in data collection on the results. Finally, a total of 10,113,560 individuals were analyzed and followed up until 2018 (Fig. 1).

### Clinical parameters and diagnostic criteria

Baseline data were collected on the following variables associated with the risk of OSAS: age (years), alcohol consumption (in 1 week and on a single occasion; 30 g/day = heavy drinkers^19^), income level, sex, and smoking status. The regular exercise has been defined as vigorous exercise at least 3 days a week or moderate exercise at least 5 days a week. BMI was calculated by dividing the subject’s weight (kilograms) by the square of the subject’s height (meters). According to the criteria for the Asia–Pacific region, participants with a BMI ≥ 25 kg/m^2^ were considered obese^20^. Diabetes mellitus (DM) was defined as International Classification of Disease 10th Revision (ICD-10) codes E11–14, plus at least one prescription of antidiabetic medication per year or a fasting glucose level ≥ 7 mmol/L (data obtained from the health database). Hypertension was indicated by a prescription (at least once a year) of a antihypertensive agent under ICD-10 codes I10–I15, or systolic blood pressure /diastolic blood pressure [SBP/DBP] ≥ 140/90 mmHg^21^. Dyslipidemia was indicated by ICD-10 code E78 along with at least one prescription of lipid-lowering agents per year, or total cholesterol ≥ 240 mg/dL^22^. OSAS was defined as G47.30 in ICD-10 code as previously reported^23^. The OSAS group comprised those with OSAS diagnosed between 2009 and 2018^24,25^. Abdominal obesity was classified based on the International Obesity Task Force Asia–Pacific region and the cutoffs for Korean adults proposed by the Korean Society for the Study of Obesity^26^.

### Statistical analyses

To calculate the incidence of OSAS, the number of obstruction events was divided by the person–time at risk. The independent association of MetS with the risk of OSAS was determined using a Cox proportional hazards model adjusted for age, sex, smoking status, alcohol consumption, regular physical exercise status, and BMI. Associations of single and combined MetS components with the risk of OSAS development were evaluated. All statistical analyses were performed using SAS software (version 9.4; SAS Institute, Cary, NC, USA) and a two-sided p-value < 0.05 was considered statistically significant.

## Results

The subjects were divided into two groups according to the presence or absence of MetS. The basic characteristics of the participants are shown in Table 1. The OSAS group was younger, male than female, less income, and higher rates of smoking and heavy drinkers. In addition, the OSAS group showed higher BMI, hypertension, dyslipidemia, higher total cholesterol, higher triglyceride, higher waist circumference, higher LDL cholesterol, and lower HDL cholesterol, indicating association with MetS. However, they exercised more regularly and had a lower incidence of DM. During 2009, 74,660 patients were newly diagnosed with OSAS (OSAS group). The OSAS group participants were younger, more likely to be male, had a lower income, and were more likely to be a current smoker, heavy drinker, and engage in regular exercise compared to the non-OSAS group. The OSAS group participants were more likely to have hypertension and dyslipidemia, and had a higher BMI, larger waist circumference, and higher levels of total cholesterol, low-density lipoprotein cholesterol (LDL-C) and TG. However, the non-OSAS group patients were more likely to have DM, and also had a lower HDL-C level. To further examine the relationship between OSAS and MetS, demographic data such as age, sex, and BMI, as well as smoking, drinking, and exercise status, were adjusted for in multivariable analysis.

**Table 1:** Baseline characteristics of patients with OSAS and controls.

	Non-OSAS	OSAS	*p*-value
	(n = 10,038,900)	(n = 74,660)	
Age (years)	47.1 ± 14.11	44 ± 11.85	< 0.0001
Sex, male (n, [%])	5,495,010 (54.74)	58,273 (78.05)	< 0.0001
Income, low 20% (n, [%])	1,757,833 (17.51)	10,228 (13.7)	< 0.0001
Current smoker (n, [%])	2,626,362 (26.16)	25,928 (34.73)	< 0.0001
Heavy drinker (≥ 30 g/day) (n, [%])	794,290 (7.91)	8675 (11.62)	< 0.0001
Regular exercise (n, [%])	1,819,385 (18.12)	14,643 (19.61)	< 0.0001
Body mass index (kg/m^2^)	23.69 ± 3.44	25.23 ± 3.33	< 0.0001
Diabetes mellitus (n, [%])	876,900 (8.74)	5307 (7.11)	< 0.0001
Hypertension (n, [%])	2,701,840 (26.91)	21,204 (28.4)	< 0.0001
Dyslipidemia (n, [%])	1,820,905 (18.14)	15,583 (20.87)	< 0.0001
Total cholesterol (mg/dL)	195.22 ± 41.46	198.56 ± 39.24	< 0.0001
Systolic blood pressure (mmHg)	122.43 ± 15.05	123.61 ± 13.86	< 0.0001
Diastolic blood pressure (mmHg)	76.3 ± 10.05	77.79 ± 9.89	< 0.0001
HDL cholesterol (mg/dL)	56.52 ± 33.05	53.03 ± 27.21	< 0.0001
LDL cholesterol (mg/dL)	121.16 ± 214.76	124.99 ± 241.95	< 0.0001
Triglyceride (mg/dL)	112.49 (112.45–112.53)	131.53 (130.98–132.08)	< 0.0001
Waist circumference (cm)	80.2 ± 9.43	84.61 ± 8.86	< 0.0001

OSAS: obstructive sleep apnea syndrome; LDL: low density lipoprotein; HDL: high density lipoprotein.

### Multivariable-adjusted analysis of the association between MetS and OSAS

**Figure 2:**
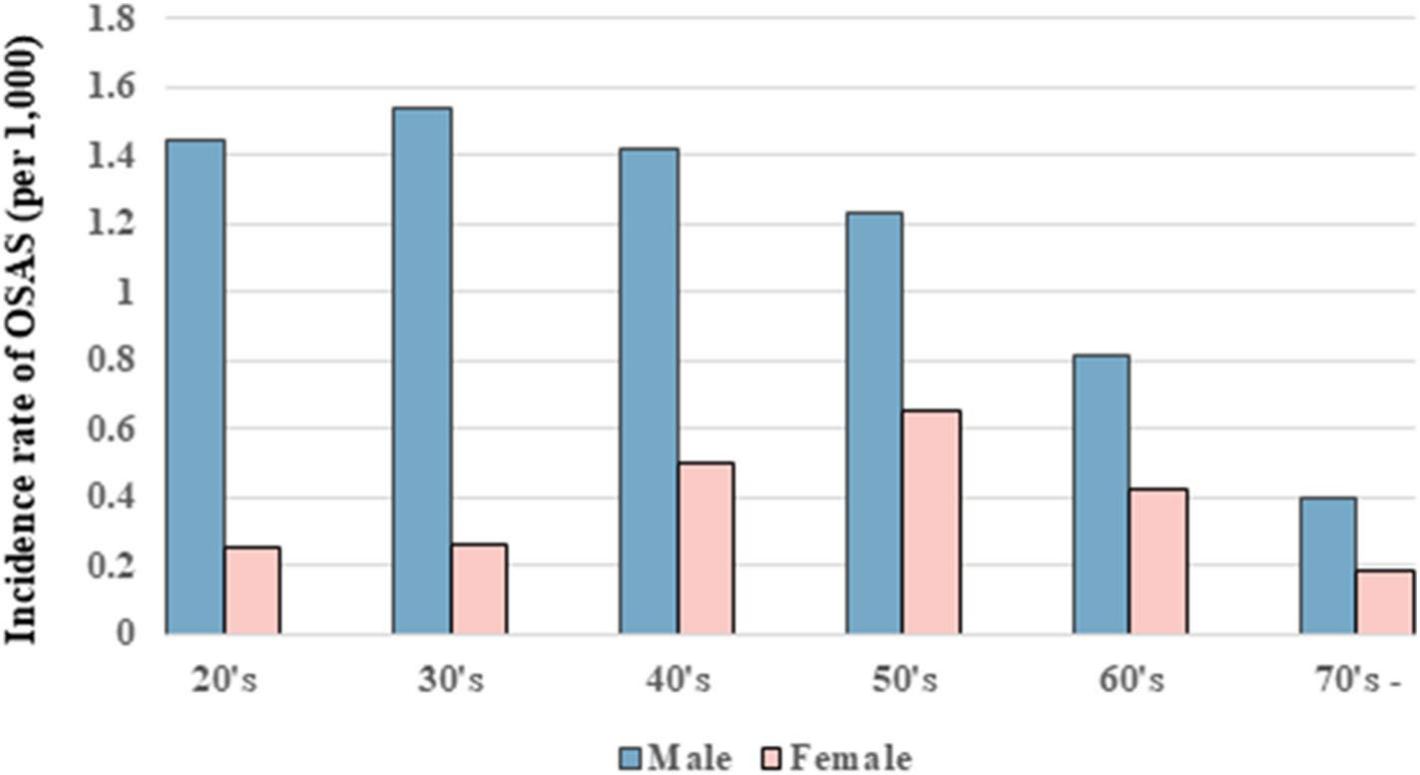
Incidence of OSAS according to age (for the 2009–2018).

**Figure 3:**
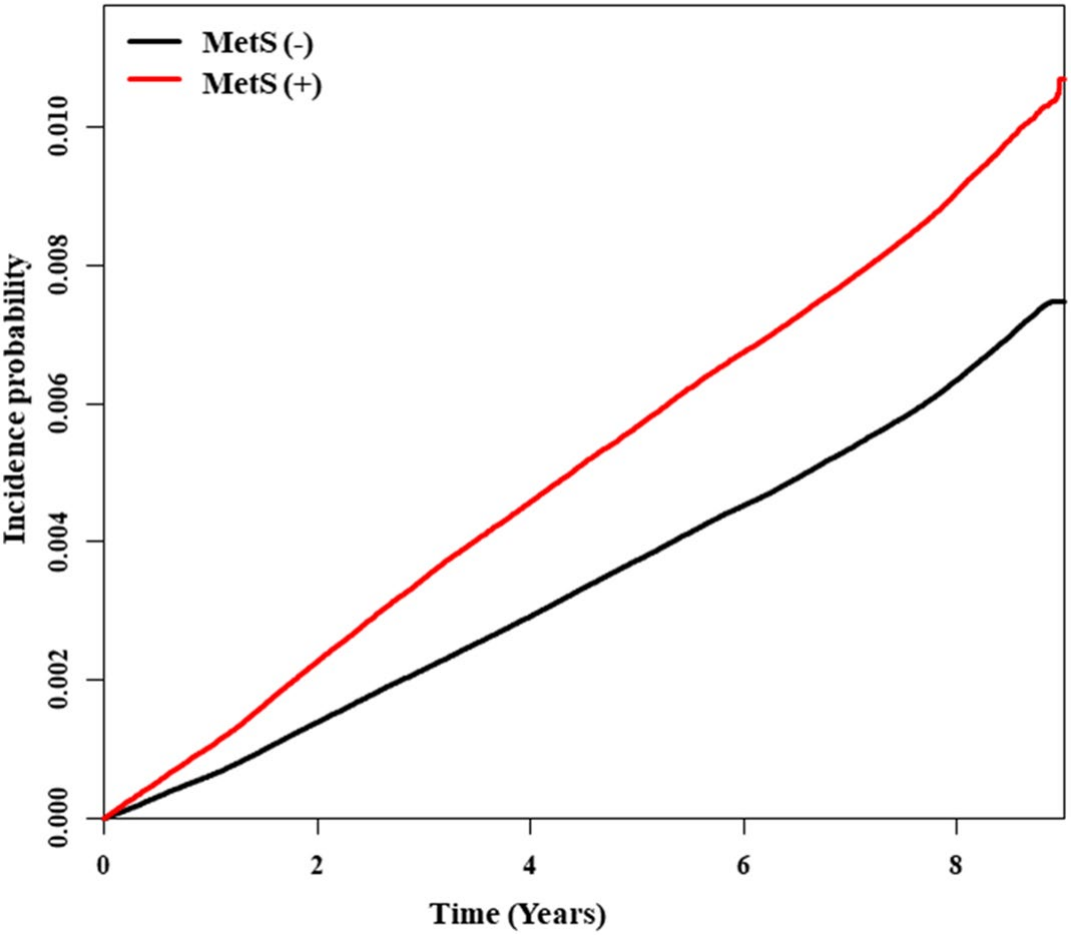
Incidence of OSA over the 10-year follow up period between MetS and non-MetS patients (log-rank test, p < 0.0001).

**Figure 4:**
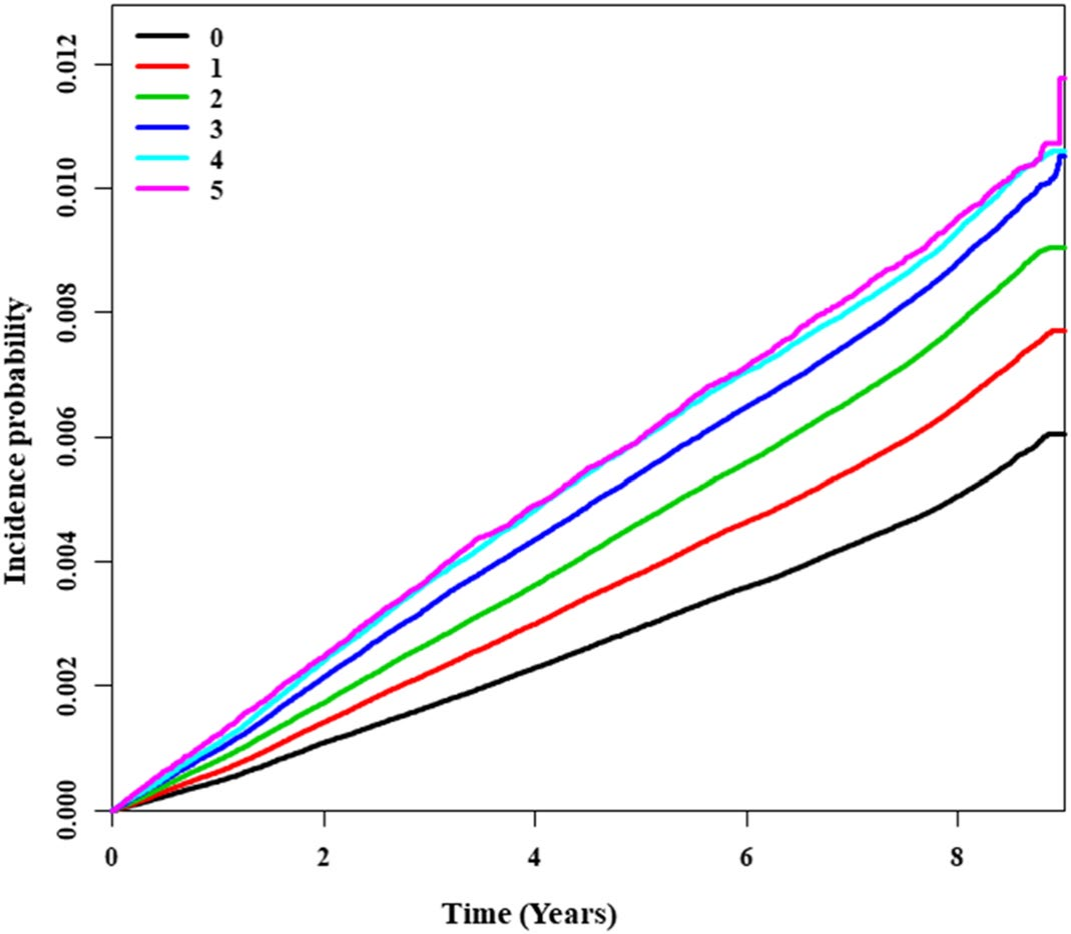
Incidence of OSA over the 10-year follow up period according to the numbers of MetS components (all components; log-rank test, p < 0.0001).

The incidence rate of OSAS in the non-MetS and MetS groups was 0.82 and 1.16, respectively (Table 2). The incidence rate of OSAS peaked in men in their 30 s and in women in their 50 s, which is when menopause begins (Fig. 2). The incidence rate of OSAS was higher in males, and the incidence probability of OSAS between the MetS and non-MetS population showed a significant difference between groups (log-rank test, p < 0.0001; Fig. 3). The hazard ratio (HR) for OSAS was 1.50 (95% confidence interval [CI] 1.52–1.57) in the MetS group. The HR of OSAS was higher in males (1.57; 95% CI 1.54–1.60) than females (1.31; 95% CI 1.26–1.36) (p < 0.0001). The HR (95% CI) for OSAS increased with the number of MetS components, being 1.25 (1.23–1.28), 1.52 (1.48–1.55), 1.83 (1.78–1.87), 2.11 (2.05–2.17) and 2.38 (2.28–2.48) for 1, 2–5 components, respectively, after adjusting for age, sex, smoking status, alcohol consumption, exercise status, and BMI. Also, the HRs were higher in men with 1–5 MetS components (1.22 [1.19–1.25], 1.46 [1.43–1.50], 1.79 [1.74–1.84], 2.12 [2.05–2.19] and 2.50 [2.38–2.62], respectively)] than in women (1.25 [1.19–1.30], 1.48 [1.41–1.56], 1.62 [1.53–1.71], 1.68 [1.58–1.79] and 1.67 [1.53–1.83], respectively) (Table 2). The probability of OSAS increased as the number of MetS components increased, and with the passage of time (log-rank test, p < 0.0001; Fig. 4).

**Table 2:** Incidence rates and HRs of OSAS according to the number of MetS components, based on an adjusted multivariable analysis (for the period 2009–2018).

	Non-OSAS	OSAS	IR	Hazard ratio (95% CI)^a^			
				Total	Male	Female	p-value^#^
**MetS**							
No^b^	7,569,064	50,823	0.82	1 (reference)	1 (reference)	1 (reference)	
Yes	2,544,496	23,837	1.16	1.50 (1.52, 1.57)	1.57 (1.54, 1.60)	1.31 (1.26, 1.36)	< 0.0001
**Number of components**							
0	2,731,043	14,760	0.65	1 (reference)	1 (reference)	1 (reference)	
1	2,732,770	18,846	0.84	1.25 (1.23, 1.28)	1.22 (1.19, 1.25)	1.25 (1.19, 1.30)	
2	2,105,251	17,217	1.00	1.52 (1.48, 1.55)	1.46 (1.43, 1.50)	1.48 (1.41, 1.56)	
3	1,440,026	13,206	1.13	1.83 (1.78, 1.87)	1.79 (1.74, 1.84)	1.62 (1.53, 1.71)	
4	822,911	7906	1.19	2.11 (2.05, 2.17)	2.12 (2.05, 2.19)	1.68 (1.58, 1.79)	
5	281,559	2725	1.20	2.38 (2.28, 2.48)	2.50 (2.38, 2.62)	1.67 (1.53, 1.83)	
≥ 3	2,544,496	23,837	1.16	1.96 (1.91, 2.00)	1.94 (1.90, 1.99)	1.64 (1.57, 1.73)	< 0.0001

IR; incidence rate (per 1,000), OSAS; obstructive sleep apnea syndrome, CI; confidence interval, MetS; metabolic syndrome.

^#^p for interaction < 0.001 between male and female.

^a^Adjusted for age, sex, smoking status, alcohol consumption, regular exercise, and body mass index.

^b^Individuals who had 0–2 of MetS components.

### OSAS risk according to combinations of metabolic syndrome components

We analyzed the incidence rates and multivariable-adjusted HRs for OSAS according to combinations of MetS components. All five components were individually associated with an increased risk of OSAS. Increases in waist circumference (HR: 1.99; 95% CI 1.90–2.08) and TG level (HR: 1.32; 95% CI 1.28–1.37) were associated with a marked increase in the risk of OSAS (Table 3). For the waist circumference component, there was a remarkable difference between men (HR: 2.00 [1.90, 2.10]) and women (HR: 1.68 [1.52, 1.86]). Among those in whom the criterion for MetS (i.e., presence of ≥ 3 of the 5 MetS components) was not met, there was a strong association of OSAS with waist circumference and TG level (HR: 2.22; 95% CI 2.10–2.34). Among patients with three MetS components, the three components associated with the highest risk of OSAS were large waist circumference, high TG level, and high HDL-C level (HR: 2.68; 95% CI 2.52–2.85). Among patients with four MetS components, the risk of OSAS was highest in those with the above combination plus hypertension (HR: 2.82; 95% CI 2.70–2.95) (Table 3).

**Table 3:** Incidence rates and HRs of OSAS according to the presence of a combination of MetS components (for the period 2009–2018).

Combination	Total	Male	Female
	HR (95% CI)^a^	HR (95% CI)+	HR (95% CI)+
Null	1 (ref.)	1 (ref.)	1 (ref.)
Glucose↑	1.08 (1.04, 1.12)	1.03 (0.99, 1.08)	1.17 (1.09, 1.26)
HBP	1.19 (1.15, 1.22)	1.13 (1.09, 1.16)	1.25 (1.17, 1.33)
WC↑	1.99 (1.90, 2.08)	2.00 (1.90, 2.10)	1.68 (1.52, 1.86)
TG↑	1.32 (1.28, 1.37)	1.27 (1.23, 1.32)	1.32 (1.19, 1.47)
HDL↓	1.16 (1.11, 1.22)	1.19 (1.11, 1.27)	1.16 (1.09, 1.24)
Glucose↑/HBP	1.06 (1.01, 1.10)	1.02 (0.98, 1.07)	1.12 (1.01, 1.23)
Glucose↑/WC↑	1.89 (1.76, 2.02)	1.86 (1.72, 2.01)	1.75 (1.49, 2.06)
Glucose↑/TG↑	1.30 (1.23, 1.37)	1.27 (1.20, 1.34)	1.28 (1.07, 1.54)
Glucose↑/HDL↓	1.11 (1.01, 1.22)	0.92 (0.80, 1.06)	1.30 (1.14, 1.47)
HBP/WC↑	2.05 (1.96, 2.14)	2.03 (1.94, 2.13)	1.68 (1.51, 1.86)
HBP/TG↑	1.41 (1.36, 1.47)	1.37 (1.32, 1.43)	1.29 (1.13, 1.48)
HBP/HDL↓	1.42 (1.33, 1.52)	1.30 (1.18, 1.42)	1.38 (1.25, 1.52)
WC↑/TG↑	2.22 (2.10, 2.34)	2.20 (2.08, 2.32)	1.58 (1.28, 1.93)
WC↑/HDL↓	2.20 (2.02, 2.41)	2.24 (2.00, 2.52)	2.00 (1.74, 2.29)
TG↑/HDL↓	1.76 (1.69, 1.83)	1.63 (1.55, 1.72)	1.82 (1.68, 1.96)
Glucose↑/HBP/WC↑	1.87 (1.78, 1.98)	1.91 (1.80, 2.02)	1.47 (1.29, 1.67)
Glucose↑/HBP/TG↑	1.20 (1.15, 1.26)	1.18 (1.12, 1.24)	1.25 (1.05, 1.47)
Glucose↑/HBP/HDL↓	1.39 (1.27, 1.53)	1.32 (1.17, 1.50)	1.28 (1.11, 1.49)
Glucose↑/WC↑/TG↑	1.92 (1.79, 2.06)	1.86 (1.73, 2.00)	2.05 (1.60, 2.63)
Glucose↑/WC↑/HDL↓	1.88 (1.62, 2.19)	1.93 (1.59, 2.34)	1.66 (1.30, 2.11)
Glucose↑/TG↑/HDL↓	1.53 (1.44, 1.62)	1.46 (1.36, 1.57)	1.53 (1.36, 1.72)
HBP/WC↑/TG↑	2.38 (2.28, 2.48)	2.35 (2.24, 2.45)	1.70 (1.44, 2.01)
HBP/WC↑/HDL↓	2.19 (2.01, 2.39)	2.31 (2.07, 2.58)	1.67 (1.44, 1.93)
HBP/TG↑/HDL↓	1.89 (1.81, 1.97)	1.80 (1.71, 1.89)	1.73 (1.60, 1.87)
WC↑/TG↑/HDL↓	2.68 (2.52, 2.85)	2.70 (2.53, 2.90)	2.08 (1.81, 2.38)
Glucose↑/HBP/WC↑/TG↑	2.03 (1.94, 2.13)	2.03 (1.93, 2.13)	1.56 (1.31, 1.86)
Glucose↑/BP↑/WC↑/HDL↓	1.81 (1.62, 2.03)	1.81 (1.56, 2.10)	1.50 (1.26, 1.79)
Glucose↑/BP↑/TG↑/HDL↓	1.61 (1.54, 1.68)	1.57 (1.49, 1.65)	1.47 (1.35, 1.61)
Glucose↑/WC↑/TG↑/HDL↓	2.37 (2.20, 2.55)	2.31 (2.12, 2.51)	2.21 (1.89, 2.57)
HBP/WC↑/TG↑/HDL↓	2.82 (2.70, 2.95)	2.94 (2.80, 3.09)	1.93 (1.76, 2.13)
All of 5 components	2.33 (2.23, 2.43)	2.43 (2.31, 2.54)	1.67 (1.53, 1.83)

^a^Adjusted for age, sex, smoking status, alcohol consumption, regular exercise, and body mass index.

## Discussion

The results of this nationwide Korean population-based study showed that MetS and the OSAS are closely related. The association was stronger in men than in women. Even when the criteria for MetS were not met, all individual MetS components were risk factors for OSAS, and the risk increased with the number of components. A large waist circumference and high TG level showed the strongest correlations with OSAS among the MetS components. Among patients with three or more MetS components, those with an elevated HDL-C and/or hypertension, accompanied by a large waist circumference and high TG level, showed a higher likelihood of OSAS.

Obesity is strongly associated with MetS and a well-known risk factor for OSAS^27^. As the proportion of obese people continues to rise, the link between OSAS and MetS has become increasingly apparent^28^. Simple obesity (based on BMI) should be adjusted for in analyses of the relationship between OSAS and MetS, as a major potential confounder^14^. Age, sex, income, smoking status, and alcohol consumption are other important confounders that should be adjusted for^14,29^, but many studies did not do this^14^. In this study, we tried to minimize confounding effects by adjusting for BMI, age, sex, smoking status, alcohol consumption, and exercise status.

Abdominal obesity is different from simple obesity. Waist circumference mainly depends on the fatty tissue in the peritoneum (i.e., in the areas between the stomach, liver, kidneys, intestines and other organs). High levels of visceral fat were also observed in obese and metabolically obese normal weight people^30^. Visceral fat is now known to be a metabolically active tissue type, in which a large amount of proinflammatory substances and vasoactivators are produced^31^; these can cause metabolic dysregulation and atherogenesis. Also, serum leptin (adipocytokines) levels are considerably higher in patients with OSAS, suggesting that OSAS might be associated with leptin resistance^32^. Leptin resistance increases the likelihood of developing OSAS^33^. In addition to leptin resistance, abdominal obesity can cause systemic inflammation and metabolic dysfunction, leading to OSAS^34^. Compared to peripheral obesity, abdominal obesity has a greater effect on upper airway function^35^. Therefore, waist circumference should be measured as an important risk factor for OSAS. Visceral fat accounts for 5–8% of total body fat in women, and increases in menopause, while in men it accounts for 10–20% of total body fat^36^. Since the prevalence of abdominal obesity is higher in men than women, waist circumference can also be considered an important factor in the difference in incidence of OSAS between genders, and might also explain why the OSAS incidence is higher in menopausal women.

Recent studies have consistently demonstrated an independent association between adult OSAS and insulin resistance^37–40^. Insulin resistance sustains a low-grade inflammatory state, which can lead to upper airway narrowing, respiratory muscle fatigue, and decreased dilator muscle contraction^34^. The low success rate of continuous positive airway pressure treatment and upper airway surgery, for improving MetS or achieving weight loss in OSAS patients, may be explained by insulin resistance^41–43^.

There is considerable evidence that OSAS can lead to MetS^44^. Repeated respiratory obstruction events in OSAS result in intermittent hypoxia and frequent arousal. Temporarily ischemic tissue can release free radicals that cause oxidative stress, cytokine release and systematic inflammation^45,46^. Also, frequent arousal causes imbalances in the sympathetic nervous system and circadian misalignment. Consequently, intermittent hypoxia and the resultant oxidative stress, sympathetic activation, and sleep fragmentation have been suggested to underlie the pathogenic links between OSAS and glucose intolerance^47^, insulin resistance^48^, hypercholesterolemia^49^ and hyperlipidemia^50^, all of which are MetS components. As shown in the literature, Mets and OSAS share various pathologic mechanisms and together constitute a potentially pathologic vicious cycle.

Our research had both strength and limitations. This is the largest population-based study to date, with the NHIS database covering the entire Korean population. However, any study that uses claims data is associated with a risk of misclassification. Therefore, potential confounding factors may exist and affect the quality of data. However, these could be compensated with large population data and high HR. Also, although causality could not be inferred in this study, a high risk of OSAS was observed among MetS patients according to our analysis of a nationwide population-based dataset covering a 10-year period.

## Conclusions

OSAS is considered a major cause of MetS, and MetS can likewise trigger the development of OSAS. In this nationwide population-based analysis adjusted for several confounding factors, we confirmed the association of MetS components with OSAS. This is important because the coexistence of two pathologies within the same patient increases the levels of biomarkers, which directly contribute to, or increase the potential for, complications. Among the MetS components, particular attention should be paid to abdominal obesity in relation to OSAS, especially in men.

## Data Availability

The data that support the findings of this study are available from the Health Insurance Review & Assessment Service (HIRA). Restrictions apply to the availability of these data, which were used under license for the current study, and so are not publicly available due to personal information protection. Data are available at https://opendata.hira.or.kr/ with the permission of the HIRA.
